# The Putative Methyltransferase *Tl*LAE1 Is Involved in the Regulation of Peptaibols Production in the Biocontrol Fungus *Trichoderma longibrachiatum* SMF2

**DOI:** 10.3389/fmicb.2020.01267

**Published:** 2020-06-12

**Authors:** Jin-Chao Shi, Wei-Ling Shi, Yan-Rong Zhou, Xiu-Lan Chen, Yu-Zhong Zhang, Xia Zhang, Wei-Xin Zhang, Xiao-Yan Song

**Affiliations:** ^1^State Key Laboratory of Microbial Technology, Marine Biotechnology Research Center, Shandong University, Qingdao, China; ^2^Laboratory for Marine Biology and Biotechnology, Qingdao National Laboratory for Marine Science and Technology, Qingdao, China; ^3^College of Marine Life Sciences, Ocean University of China, Qingdao, China; ^4^Department of Molecular Biology, Qingdao Vland Biotech Inc., Qingdao, China

**Keywords:** *Trichoderma longibrachiatum* SMF2, LAE1, conidiation, peptaibol, secondary metabolite

## Abstract

The biocontrol fungus *Trichoderma longibrachiatum* SMF2 secretes a large quantity of peptaibols that have been shown to have a range of biological activities and therefore great application values. However, the mechanism of the regulatory expression of peptaibols is still unclear. The putative methyltransferase LaeA/LAE1 is a global regulator involved in the biosynthesis of some secondary metabolites in filamentous fungi. In this study, we demonstrated that the ortholog of LaeA/LAE1 in the biocontrol fungus *T. longibrachiatum* SMF2, *Tl*LAE1, plays an important role in the regulation of peptaibols production. Deletion of *Tllae1* resulted in a slight negative impact on mycelial growth, and a significant defect in conidial production. Deletion of *Tllae1* also compromised the production of peptaibols to a large degree. Further analyses indicated that this defect occurred at the transcriptional level of the two synthetases-encoding genes, *tlx1* and *tlx2*, which are responsible for peptaibols production. By contrast, constitutive expression of *Tllae1* in *T. longibrachiatum* SMF2 led to 2-fold increased peptaibols production, suggesting that this is a strategy to improve peptaibols production in *Trichoderma* fungi. These results demonstrate the important role of LAE1 in the regulation of peptaibols production in *T. longibrachiatum* SMF2.

## Introduction

Peptaibols are a group of small peptides containing 5–20 amino acid residues, some of which are non-proteinogenic amino acids. They are characterized as having a high content of α-Aminoisobutyric acid (Aib) residues and generally have both N-terminal (mainly acetyl groups) and C-terminal modifications in the form of amino alcohol groups rather than amino acids. Peptaibols are ecologically and commercially important due to their antimicrobial and anticancer properties as well as their ability to induce systemic resistance in plants against microbial invasion (Wiest et al., 2002; Viterbo et al., 2007; Reino et al., 2008).

Fungal species of the genus *Trichoderma*, such as the well characterized *T. atroviride*, *T. virens*, and *T. longibrachiatum*, are excellent producers of peptaibols (Marik et al., 2019). Of these fungal producers, *T. longibrachiatum* SMF2 produces a large quantity of peptaibols named as Trichokonins (TKs) that are mainly classified into 20-aa Trichokonins A (TKA) and 12-aa Trichokonins B (TKB) (Song et al., 2006; Xie et al., 2014). TKs produced by *T. longibrachiatum* SMF2 have been demonstrated to have broad-spectrum antimicrobial activities (Song et al., 2006; Shi et al., 2012) as well as other biological functions including the elicitation of systemic resistance in tobacco and Chinese cabbage (Luo et al., 2010; Li et al., 2014) and the induction of programmed cell death in tumor cells (Shi et al., 2010).

Fungal peptaibols are naturally synthesized by large multi-module protein complexes known as non-ribosomal peptide synthetases (NRPSs), in which each module catalyzes the incorporation of a single proteinogenic or non-proteinogenic amino acid (El-Bondkly, 2014). Combining genome sequencing and targeted gene deletion analyses, the two NRPS encoding genes, *tlx1* and *tlx2*, responsible for 20-aa TKA and 12-aa TKB, have been identified in *T. longibrachiatum* SMF2 (Xie et al., 2015; Zhou et al., 2019), which is consistent with the reports that two peptaibol synthases are present in the genomes of *T. longibrachiatum* and other 11 different *Trichoderma* spp. (Kubicek et al., 2019). We recently demonstrated that the *stp1* gene, encoding a putative glucose sensor in *T. longibrachiatum* SMF2, represses peptaibols production, and *stp1* deletion led to significantly enhanced expression of *tlx1* and *tlx2*, and therefore remarkably increased peptaibols production (Zhou et al., 2019). Despite this, our understanding of the regulatory mechanism of peptaibols production in *T. longibrachiatum* is much limited, which hampers efficient genetic engineering for construction of hyperproducing strains.

The putative S-adenosylmethionine-dependent methyltransferase LaeA (stands for “loss of aflR expression-A”), which forms a trimeric protein complex with VeA and VelB, has been demonstrated to be involved in the biosynthesis of a large number of secondary metabolites in many filamentous fungi, such as *Aspergillus nidulans*, *Penicillium chrysogenum*, *Fusarium fujikuroi*, and *Cochliobolus heterostrophus* (Bok and Keller, 2004; Bok et al., 2006; Kale et al., 2008; Hoff et al., 2010; Wiemann et al., 2010; Oda et al., 2011; Wu et al., 2012). LaeA is also involved in the regulation of conidiation in many filamentous fungi (Sugui et al., 2007; Bayram et al., 2010; Hoff et al., 2010; Wiemann et al., 2010; Chang et al., 2012; Jiang et al., 2012; Wu et al., 2012) and fruiting body formation in *Aspergillus* species (Amaike and Keller, 2009). LaeA orthologs, named LAE1, have been found in *Trichoderma* species including *T. reesei* and *T. atroviride*. LAE1 regulates the expression of cellulases and polysaccharide hydrolases in *T. reesei* (Seiboth et al., 2012), which is an excellent cellulolytic fungus widely applied in industry (Bischof et al., 2016; Druzhinina and Kubicek, 2016). Transcriptome analyses on *lae1*-null and -overexpressing *T. reesei* strains were further performed to assess the role of LAE1 in genome-wide gene expression. Genes significantly regulated by LAE1 include those encoding ankyrin proteins, iron uptake, heterokaryon incompatibility proteins, PTH11-receptors, and oxidases/monooxygenases (Karimi-Aghcheh et al., 2013). In addition, 7 of 17 polyketide or non-ribosomal peptide synthase encoding genes are positively regulated by LAE1 (Karimi-Aghcheh et al., 2013). Notably, one NRPS synthetase encoding gene responsible for synthesis of paracelsin, one kind of peptaibol secreted by *T. reesei* (Neuhof et al., 2007), is significantly up-regulated in the *lae1* null mutant but also *lae1*-overexpressing mutant (Karimi-Aghcheh et al., 2013). Moreover, similar to orthologs in other filamentous fungi, LAE1 in *T. reesei* and *T. atroviride* are found to be positively involved in conidiation (Aghcheh et al., 2013; Karimi-Aghcheh et al., 2013).

In this study, we presented that the ortholog of LaeA/LAE1 in the biocontrol fungus *T. longibrachiatum* SMF2, *Tl*LAE1, is involved in the regulation of peptaibols production. The results showed that targeted deletion of *Tllae1* resulted in a significant defect in peptaibols production, whereas constitutive overexpression of *Tllae1* evidently enhanced the yield of peptaibols in *T. longibrachiatum* SMF2.

## Materials and Methods

### Strains, Media and Cultivation Conditions

*Trichoderma longibrachiatum* SMF2 (CCTCC No. 209031) stocked in our laboratory was used as the wild type (WT) strain. *T. longibrachiatum* SMF2 strains were routinely maintained on potato dextrose agar (PDA) plates. *Escherichia coli* DH5α was used for plasmid construction and cloning.

### Construction of *T. longibrachiatum* SMF2 Recombinant Strains

An overlap extension PCR method (Amaike and Keller, 2009) was used to create the fragments for targeted gene deletion. Two approximately 2.0 kb fragments upstream and downstream of *Tllae1* were amplified from the genomic DNA of *T. longibrachiatum* SMF2 using the primer pairs Δ*Tllae1*upF/Δ*Tllae1*upR and Δ*Tllae1*downF/Δ*Tllae1*downR, respectively. The 2.4 kb *hph* gene (a hygromycin B phosphotransferase encoding gene) was amplified using the primer pair FhygBF/FhygBR from the plasmid pUCATPH (Lu et al., 1994). The three resulting fragments were fused and amplified using the primer pair CΔ*Tllae1*F/CΔ*Tllae1*R, and was subsequently transformed into *T. longibrachiatum* SMF2 to construct the *Tllae1-*deleted mutant Δ*Tllae1*. To generate the complementation vector for *Tllae1*, three fragments named CF1, CF2, and CF3 were amplified individually and ligated into the plasmid pMD-19T. Specifically, the putative native promoter (∼1.0 kb), full-length coding sequence (∼2.2 kb), and the putative terminator of *Tllae1* (∼0.5 kb) were amplified from *T. longibrachiatum* SMF2 with primers Cp*ΔTllae1*upF/Cp*ΔTllae1*upR to generate the fragment CF1. The second fragment CF2 (∼3 kb), the acetamidase-encoding gene (*amdS*) cassette, was amplified from the pALK424 vector using the primer pair CpΔ*Tllae1-amdS*F/CpΔ*Tllae1-amdS*R. The third fragment CF3 (∼1.6 kb) which is immediately downstream *Tllae1* coding sequence was amplified from *T. longibrachiatum* SMF2 genomic DNA using the primer pair CpΔ*Tllae1*downF/CpΔ*Tllae1*downR. The pMD-19T plasmid containing the above three fragments was linearized with *Hin*dIII and applied to transform the Δ*Tllae1* cells to generate the *Tllae1* complementation strain. To obtain the overexpression vector, the fragment including the full-length coding sequence of *Tllae1* and its native terminator was amplified from *T. longibrachiatum* SMF2 genomic DNA with primers *Tllae1*CE-F/*Tllae1*CE-R, and was ligated into the pIG1783 plasmid (Penttilä et al., 1987), which contains the promoter sequence of the glyceraldehydes- 3-phosphate dehydrogenase encoding gene (*gpd*). The resultant plasmid pIG1783-*Tllae1*CE was linearized with *Hin*dIII, and then transformed the *T. longibrachiatum* SMF2 cells to generate the *Tllae1* overexpression strain.

Transformation of *T. longibrachiatum* SMF2 was carried out essentially as previously described (Zhou et al., 2012). For southern blot hybridization, genomic DNA extracted from the WT or Δ*Tllae1* strain was digested using *Sma*I prior to hybridization. Detection of probe-hybridized DNA fragment was carried out using the DIG High Prime DNA Labeling and Detection Starter Kit I (Roche Diagnostics, Mannheim, Germany). The primers used in this study were listed in Supplementary Table S1. The illustration of recombinant strain construction and verification was shown in Supplementary Figures S1–S3.

### Vegetative Growth, Conidiation and Conidial Germination Analyses

To compare the vegetative growth of the WT and mutant strains, a same-size piece (1-cm-diameter) of mycelia of each strain were inoculated on PDA plates and incubated at 28°C for 7 days. To analyze biomass accumulation, equal amounts of conidia (2 × 10^6^) were inoculated into 100 mL of potato dextrose broth (PDB) (Hermosa et al., 2004) and cultured at 28°C. Mycelia were harvested at an interval of 24 h, and the dry weight of mycelia after dehydration at 65°C was determined. To analyze conidiation, approximately 10^4^ conidia were spread on the 90-mm PDA plates and incubated under constant white light (31 μmol photons m^–2^s^–1^, 2200 lx) condition at 28°C for 3 days. The number of conidia was counted with a hemocytometer under an inverted optical microscope (Olympus, Tokyo, Japan). To analyze conidial germination, approximately 10^4^ conidia were inoculated into 100 mL of PDB, and conidial germination after 12 h-cultivation was analyzed under an optical microscope.

### Hydrophobicity Assay

Approximately 10 μL of the conidia suspension (10^6^ conidia/mL) of all the tested strains was inoculated onto PDA plates and cultivated at 28°C for 7 days. Fifty μL of water was placed on a fungal colony of each strain and maintaining of the drop of water on the colony surface was monitored.

### Scanning Electron Microscope Observation

To visualize the hyphal morphology difference between the mutant and WT strains, equal amounts of approximately 10^8^ conidia were inoculated into PDB medium and cultured at 28°C for 48 h. Mycelia were fixed with 2.5% glutaraldehyde in 100 mM PBS buffer (pH 7.2) at 4°C for 12 h, and then washed with 100 mM PBS buffer three times. They were further fixed with 1% (w/v) osmium tetroxide for 2 h at room temperature, followed by wash with 100 mM PBS buffer three times, and then dehydrated with ethanol. Samples were mounted, sputter coated with 60% gold and 40% palladium, and finally viewed with a JEOL JSM-7600F scanning electron microscope.

### High-Performance Liquid Chromatography (HPLC) Analyses

High-Performance Liquid Chromatography analyses were performed to quantify the yield of peptaibols production essentially as previously described (Zhou et al., 2019). Approximately equal amounts of approximately 10^8^ conidia of the WT and mutant strains were inoculated into 500 mL Erlenmeyer flasks containing 100 mL PDB, and were cultivated at 28°C for 10 days with shaking at 160 rpm. After centrifugation at 12,000 rpm for 20 min, 30 mL of the collected culture supernatant was loaded on a Cleanert C_18_ SPE cartridges (500 mg/6 mL, Agela Technologies, China) and eluted with 2 mL methanol to achieve 15 times concentrated peptaibol samples. The samples (15 μL each) were subsequently subjected to HPLC to analyze the content of peptaibols using a reversed phase analytical column (Sunfire C18, 4.6 × 250 mm, Waters, Ireland). The solvent system was MeOH/H_2_O (84:16, v/v) at a flow rate of 1.0 mL min^–1^ and the chromatogram was monitored at 203 nm (Wiest et al., 2002; Mukherjee et al., 2011). The fungal mycelia after different incubation periods were collected, dried and weighted. Peptaibols collected previously (Zhou et al., 2019) were used as standards. The production yield of peptaibols was determined as the peptaibols amount per mg fungal biomass.

### Quantitative Real-Time RT-PCR

Wild type, Δ*Tllae1*, CpΔ*Tllae1* and CE*Tllae1* strains were cultivated in PDB at 28°C for 48 h. Total RNA was extracted from the harvested mycelia using TRIzol reagent (Invitrogen, Grand Island, NY, United States) and further treated with the TURBO DNA-free kit (Ambion, Austin, TX, United States) to remove genomic DNA according to the manufacturer’s instruction. Reverse transcription was carried out using the PrimeScript RT reagent kit (Takara, Tokyo, Japan) according to the instruction. Quantitative PCR was performed on a LightCycler 480 II thermocycler (Roche, Basel, Switzerland). Amplification reactions were performed using the SYBR Green Supermix (Takara, Tokyo, Japan) according to the manufacturer’s instructions. Data analysis was performed using the comparative CT method (Schmittgen and Livak, 2008). The endogenous *tef1* gene was used as the control for normalization (Brunner et al., 2008). Primers are listed in Supplementary Table S1.

### Sequence Analysis

The amino acid sequences of proteins were retrieved from NCBI or JGI databases. Sequence alignments were performed using Clustal W (Thompson et al., 2002). Phylogenetic tree was constructed with MEGA7.0 (Kumar et al., 2016) using the neighbor-joining method with 1,000 bootstraps.

### Statistical Analysis

Statistical analysis was performed using the student’s *t*-test analysis. At least three biological replicates were performed for each analysis and the results and errors are the mean and SD, respectively, from three replicates.

## Results

### Identification of the LaeA/LAE1 Ortholog in *T. longibrachiatum* SMF2

**FIGURE 1:**
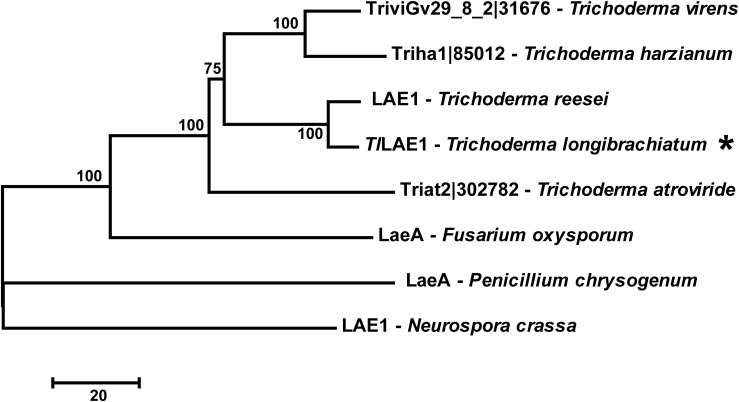
Phylogenetic analyses of *Tl*LAE1 and its orthologs. Sequence alignments were performed with Clustal W (Thompson et al., 2002), and the neighbor-joining tree was generated with Mega 7.0 (Kumar et al., 2016). Numbers on the tree branches represent the bootstrap support calculated per 1000 bootstrap replicates. JGI entry numbers of *Tl*LAE1 orthologs were shown. *Tl*LAE1 is indicated by an asterisk.

To identify the LaeA/LAE1 ortholog in *T. longibrachiatum* SMF2, its genome was searched with the amino acid sequence of *A. nidulans* LaeA (Bok and Keller, 2004) and *T. reesei* LAE1 (Seiboth et al., 2012) as inquiry, respectively, and the protein-encoding gene SMF2FGGW_101365, which was hereafter named *Tllae1*, was retrieved. The cDNA sequence of *Tllae1* has 1077 bp nucleotides in length and encodes a putative protein of 358 amino acids. The 75–260 amino acids specify the S-adenosylmethionine-dependent methyltransferase domain. Amino acid sequence comparison showed that *Tl*LAE1 shares sequence identity of 29 and 91% with LaeA/LAE1 from *A. nidulans* and *T. reesei*, respectively. Phylogenic analysis further revealed that orthologs of *Tl*LAE1 are widely distributed across filamentous ascomycete fungi, including several *Trichoderma* biocontrol fungi which produce peptaibols (Figure 1).

### Deletion of *Tllae1* Resulted in a Slight Impact on Vegetative Growth but Significantly Compromised Conidiation of *T. longibrachiatum* SMF2

**FIGURE 2:**
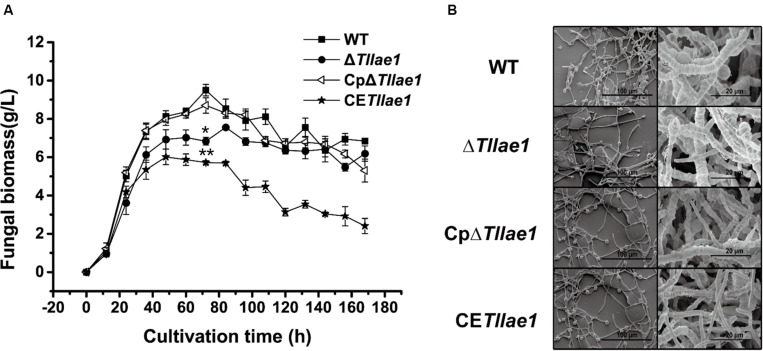
Growth analyses of *T. longibrachiatum* SMF2 and its mutants. **(A)** Determination of biomass accumulation of WT, Δ*Tllae1*, CpΔ*Tllae1* and CE*Tllae1* strains that were cultured in PDB at 28°C. Significant differences (*T*-test *P < 0.05, **P < 0.01) were observed in the biomass accumulation between WT and Δ*Tllae1*, and CE*Tllae1*. Values represent the mean of three biological replicates. Error bars are the SD from these replicates. **(B)** Scanning electron microscope observation of hyphal morphology of WT, Δ*Tllae1*, CpΔ*Tllae1* and CE*Tllae1* strains that were cultured in PDB at 28°C for 48 h. The data shown in the graph are representative of results of triplicate experiments with three independent transformants.

Targeted deletion of *Tllae1* in *T. longibrachiatum* SMF2 was performed by gene replacement of *Tllae1* coding region with an expression cassette of the hygromycin B phosphotransferase encoding gene, *hph*, and therefore, the mutant Δ*Tllae1* was constructed. To analyze the effect of the *Tl*LAE1 absence on the vegetative growth of *T. longibrachiatum* SMF2, Δ*Tllae1* and WT strains were cultured in PDB, and their biomass was determined. As shown in Figure 2A, Δ*Tllae1* displayed a slight defect in biomass accumulation, as demonstrated by a 20–30% decrease in biomass accumulation. This slight growth defect was almost completely rescued by the *in situ* complementation of *Tllae1*, whose expression was driven under its native promoter (Supplementary Figure S2). In line with the slight difference in biomass accumulation, no evident difference was observed in the mycelial morphology between the mutant and the WT strain (Figure 2B), suggesting that the absence of *Tl*LAE1 did not have a significant impact on fungal growth.

**FIGURE 3:**
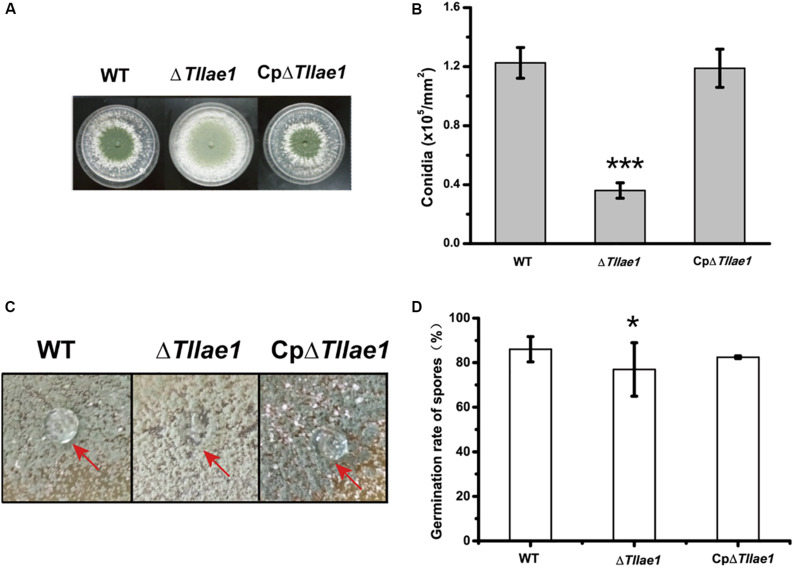
Effect of deletion of *Tllae1* on conidial production and conidial germination of *T. longibrachiatum* SMF2 and its mutants. **(A)** Phenotypic analyses of conidial production of WT, Δ*Tllae1*, and CpΔ*Tllae1* strains cultured on PDA plates at 28°C for 3 days. **(B)** Quantitation of conidia produced by WT, Δ*Tllae1*, and CpΔ*Tllae1* strains as shown in panel **(A)**. **(C)** Hydrophobic phenotypes of conidia produced by mycelial colonies of WT, Δ*Tllae1*, and CpΔ*Tllae1* strains cultured on PDA plates at 28°C for 5 days. Fifty μL of water was pipetted on the colony surface and photographs were taken 5 min later. **(D)** Analyses of conidial germination from WT, Δ*Tllae1*, and CpΔ*Tllae1* strains after being inoculated into PDB and cultured at 28°C for 12 h. Values represent the mean of three biological replicates. Error bars are the SD from these replicates. Significant differences (*T*-test *P < 0.05, ***P < 0.001) were observed in conidia production and germination between WT and Δ*Tllae1*, and CE*Tllae1*.

In contrast to mycelial growth, Δ*Tllae1* displayed a severe defect in conidiation, as evidenced by the significantly decreased formation of conidia compared to that of the WT strain (Figures 3A,B). In filamentous fungi, small secreted proteins known as hydrophobins are mostly found in conidia, making the conidia surface hydrophobic (Linder et al., 2005). We therefore examined the hydrophobicity of the mutant and WT colonies, respectively, and found that water droplets dispersed immediately on the mutant colony (Figure 3C), indicating that the mutant colony was less hydrophobic than the WT colony. This is consistent with the notion that the Δ*Tllae1* cells produced much less conidia than the WT cells. The defect in conidia production was rescued in the complemented strain, CpΔ*Tllae1* (Figure 3C). However, although the conidia production was significantly compromised, conidial germination was slightly impacted in the Δ*Tllae1* mutant (Figure 3D).

### Deletion of *Tllae1* Significantly Compromised Peptaibols Production of *T. longibrachiatum* SMF2

**FIGURE 4:**
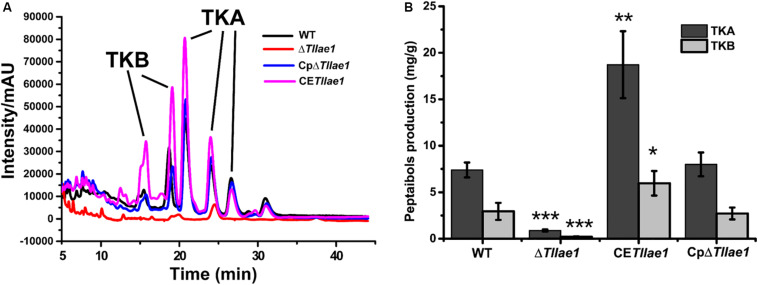
HPLC analyses of peptaibols production of *T. longibrachiatum* SMF2 and its mutants. **(A)** HPLC analyses of extracellular peptaibols produced by WT, Δ*Tllae1*, CpΔ*Tllae1*, and CE*Tllae1* strains cultured in PDB medium at 28°C for 10 days. **(B)** Peptaibols production of WT, CE*Tllae1* and CpΔ*Tllae1* strains after cultivation in PDB at 28°C. Values represent the mean of three biological replicates. Error bars are the SD from these replicates. Significant differences (*T*-test *P < 0.05, **P < 0.01, ***P < 0.001) were observed in production of TKA and TKB between WT and Δ*Tllae1*, and CE*Tllae1*.

**FIGURE 5:**
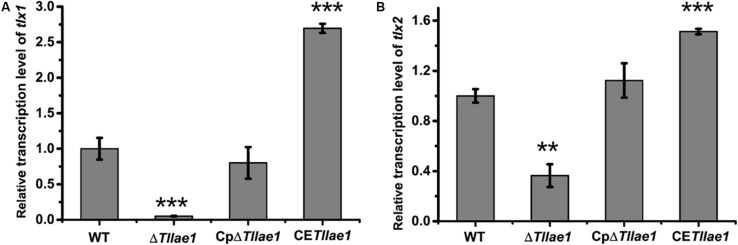
The effect of the absence or constitutive expression of *Tllae1* on the relative transcriptional levels of the two synthetases-encoding genes, *tlx1* and *tlx2*. **(A)** Relative transcription level of *tlx1*. **(B)** Relative transcription level of *tlx2*. All the strains were cultured at 28°C for 48 h. The gene *tef1* was used as the control. Values represent the mean of three biological replicates. Error bars are the SD from these replicates. Significant differences (*T*-test **P < 0.01, ***P < 0.001) were observed in the relative transcriptional levels of *tlx1* and *tlx2* between WT and Δ*Tllae1*, and CE*Tllae1*.

In order to study the role of *Tl*LAE1 in the regulation of peptaibols synthesis, we measured the extracellular amount of peptaibols in the Δ*Tllae1* and WT strains that were cultured in PDB for 10 days. As shown in Figure 4, deletion of *Tllae1* resulted in a significant defect in the production of extracellular peptaibols, compared to that of the WT strain. We further analyzed the amount of the two major peptaibols, 20-aa TKA and 12-aa TKB, and found that their synthesis was both significantly compromised, with a more severe impact on TKA. To analyze whether this effect occurred at the transcriptional level, quantitative RT-PCR was performed to determine the relative transcripts of the two peptaibol synthesase encoding genes, *tlx1* and *tlx2*, that are responsible for synthesizing TKA and TKB, respectively (Xie et al., 2014; Zhou et al., 2019). The results indicated that the relative transcription of *tlx1* and *tlx2* were both significantly decreased, with a more severe impact on that of *tlx1* (Figure 5), which is consistent with the observation that extracellular TKA production was more affected than that of TKB. The significant defect in peptaibols production resulted from *Tllae1* deletion is almost completely rescued in the complemented strain (Figures 4, 5). As a whole, the above results suggested that *Tl*LAE1 is involved in the regulation of peptaibols production.

### Constitutive Expression of *Tllae1* Led to Enhanced Peptaibols Production

We further analyzed the effect of constitutive expression of *Tllae1*, under control of the constitutive *gpd* promoter (Punt et al., 1990; Supplementary Figure S3), on extracellular peptaibols production and mycelial growth. The overexpression of *Tllae1* resulted in a compromised biomass accumulation and differing mycelial morphology (Figure 2), however, the overall peptaibols production in the overexpression strain was obviously enhanced (Figures 4, 5). Specifically, compared to those in the WT strain, production of TKA and TKB increased around 2.5- and 2-fold, respectively (Figure 4). These results indicated that increasing *Tllae1* expression is an effective strategy to improve peptaibols production in *T. longibrachiatum* SMF2.

## Discussion

LaeA was first identified as a regulator of secondary metabolism in *Aspergillus*, and later on was demonstrated to be required for the biosynthesis of many secondary metabolites in fungi (Bok and Keller, 2004; Bok et al., 2006; Kale et al., 2008; Hoff et al., 2010; Wiemann et al., 2010; Oda et al., 2011; Wu et al., 2012). Moreover, LaeA/LAE1 has been found to be involved in the regulation of conidiation and morphological development (Sugui et al., 2007; Amaike and Keller, 2009; Bayram et al., 2010; Wiemann et al., 2010; Chang et al., 2012; Jiang et al., 2012; Wu et al., 2012; Karimi-Aghcheh et al., 2013). Therefore, LaeA/LAE1 is considered to act as a global regulator. In this study, we identified the ortholog of *A. nidulans* LaeA and *T. reesei* LAE1, *Tl*LAE1, in the biocontrol fungus *T. longibrachiatum* SMF2 that produces large quantities of peptaibols. Our results showed that *Tl*LAE1 is involved in the regulation of conidiation and peptaibols production in *T. longibrachiatum* SMF2. These results are consistent with the previous reports regarding the function of LaeA/LAE1 in conidiation and secondary metabolism (Bok and Keller, 2004; Bok et al., 2006; Kale et al., 2008; Hoff et al., 2010; Wiemann et al., 2010; Oda et al., 2011; Wu et al., 2012).

It is not uncommonly observed that overexpression of LaeA results in enhanced production of several secondary metabolites in fungi. For example, several PKS (polyketide synthetase) and NRPS encoding genes have been shown to be upregulated in the *lae1*-overexpressing *T. reesei* (Karimi-Aghcheh et al., 2013); overexpression of LaeA in *A. fumisynnematus* significantly increased the production of cyclopiazonic acid, which was previously known as mycotoxin (Hong et al., 2015); and in *P. citrinum*, overexpression of LaeA enhanced mevastatin production (Zheng et al., 2014). More strikingly, overexpression of LaeA has even led to synthesis of new metabolites that have not been detected in the WT strain (Karimi-Aghcheh et al., 2013; Hong et al., 2015; Jiang et al., 2016; Soukup et al., 2016). In our study, constitutive overexpression of *Tllae1* markedly enhanced the peptaibols yield in *T. longibrachiatum* SMF2. The yield of the two main 20-aa TKA and 12-aa TKB were increased 2.5- and 2-fold, respectively, as a result of constitutive overexpression of *Tllae1*. These findings support that overexpression of LaeA/LAE1 serves as an effective strategy to improve production of biologically active secondary metabolites and even to discover new metabolites via activating silent gene clusters in fungi (Soukup et al., 2016), which are rich resources for valuable secondary metabolites that have great potentials in agriculture, industry, and pharmaceutics.

LaeA in *Aspergillus* spp. is postulated to serve as a histone methyltransferase to modify chromatin structure thereby to regulate transcription of target genes in a larger genomic region. Strauss and Reyes-Dominguez (2011) provided evidence to show that LaeA somehow counteracts the trimethylation of H3K9 and the binding of heterochromatin protein to this repressive chromatin mark (Reyes-Dominguez et al., 2010). However, in the filamentous fungus *T. reesei*, no correlation between the LAE1-modulated expression of genes and changes in histone methylation has been observed (Seiboth et al., 2012; Karimi-Aghcheh et al., 2013). This might be caused by the less conservation between LaeA from *Aspergillus* spp. and LAE1 from *T. reesei*, as demonstrated by the observation that functional *T. reesei* LAE1 does not complement an *A. nidulans* Δ*laeA* strain (Karimi-Aghcheh et al., 2013). Currently, the direct target substrates of LaeA/LAE1 are unknown, although LaeA/LAE1 was demonstrated to be a *bona fide* methyltransferase that methylates itself (Patananan et al., 2013) and is localized to the nucleus (Reyes-Dominguez et al., 2010). It is therefore speculated that LaeA/LAE1 may bind to other proteins and exert its function indirectly to control gene expression.

*Tl*LAE1 of *T. longibrachiatum* SMF2 shares high sequence identity of 90% with *T. reesei* LAE1, suggesting that these two proteins are highly conserved. Nevertheless, difference in the effect of LAE1 perturbation or overexpression on the expression of peptaibols synthetase encoding genes has been observed between *T. reesei* and *T. longibrachiatum* SMF2. One of the two peptaibol synthetase encoding genes in *T. reesei* (Trire2:23171) is up-regulated in response to LAE1 perturbation or overexpression when cells were cultivated with lactose as the sole carbon source (Karimi-Aghcheh et al., 2013), whereas the two peptaibol synthetase encoding genes in *T. longibrachiatum* SMF2 were significantly down-regulated in *lae1*-null mutant and exhibited contrasting expression pattern in *lae1*-overexpressing mutant. This discrepancy may be caused by the intrinsic contrasting functions of LAE1 in controlling the expression of the NRPS encoding genes responsible for peptaibol synthesis. However, given that the regulatory effect of LAE1 in *T. reesei* on secondary metabolism including peptaibols production appears to be growth rate dependent (Fekete et al., 2014), possibility could not be excluded that different cultivation conditions and thus different growth rates of *T. reesei* (with lactose as the sole carbon source) and *T. longibrachiatum* SMF2 (with glucose as the sole carbon source) result in this discrepancy.

We previously found that peptaibols produced by *T. longibrachiatum* SMF2 exhibited antimicrobial activity against a range of Gram-positive bacterial and fungal phytopathogens (Song et al., 2006), so it could be assumed that the elimination of LAE1 in this fungus, which caused a significant defect in peptaibols production, should remarkably affect the ability of this fungus to repress plant pathogens that is contributed by peptaibols. Moreover, given that LAE1/LaeA is considered to be a global regulator in many filamentous fungi, elimination of *Tl*LAE1 in *T. longibrachiatum* SMF2 would have broad effects on expression of a number of genes, probably including cellulase encoding genes, as the case in the well-known cellulolytic fungus, *T. reesei* (Seiboth et al., 2012). These relevant issues would be investigated in the future.

## Data Availability Statement

All datasets generated for this study are included in the article/Supplementary Material.

## Author Contributions

J-CS, W-LS, and Y-RZ performed the experiments. X-YS, W-XZ, X-LC, Y-ZZ, and XZ performed the data analysis. X-YS, Y-ZZ, and X-LC designed and supervised the project. X-LC, W-XZ, Y-RZ, and XZ wrote the manuscript. All the authors approved the manuscript.

## Conflict of Interest

XZ was employed by Qingdao Vland Biotech Inc. The remaining authors declare that the research was conducted in the absence of any commercial or financial relationships that could be construed as a potential conflict of interest.
