# Supplementary material for: The Putative Methyltransferase TlLAE1 Is Involved in the Regulation of Peptaibols Production in the Biocontrol Fungus Trichoderma longibrachiatum SMF2
Source: Front Microbiol. 2020 Jun 12;11:1267. doi: 10.3389/fmicb.2020.01267 (PMC7307461; doi:10.3389/fmicb.2020.01267)
Supplement: Supplementary file 1 [file Presentation_1.pdf]

The Putative Methyltransferase *Tl*LAE1 is Involved in the Regulation of Peptaibols Production in the Biocontrol Fungus *Trichoderma longibrachiatum* SMF2

Jin-Chao Shi[1#], Wei-Ling Shi[1#], Yan-Rong Zhou[1#], Xiu-Lan Chen[1], Yu-Zhong Zhang[1,2,3], Xia Zhang[4*], Wei-Xin Zhang[1*], Xiao-Yan Song[1*]

[1]State Key Laboratory of Microbial Technology, Marine Biotechnology Research Center, Shandong University, Qingdao, 266237, China.

[2]Laboratory for Marine Biology and Biotechnology, Qingdao National Laboratory for Marine Science and Technology, Qingdao 266237, China.

[3]College of Marine Life Sciences, Ocean University of China, Qingdao 266003, China.

[4]Department of Molecular Biology, Qingdao Vland Biotech Inc., Qingdao, China

[#]Jin-Chao Shi, Wei-Ling Shi, and Yan-Rong Zhou contributed equally to this work.

[*]Correspondence to Xiao-Yan Song, or Wei-Xin Zhang, State Key Laboratory of Microbial Technology, Shandong University, Qingdao 266237, China. Tel, 86-532-58632578; Fax, 86-532-58632578; E-mail, xysong@sdu.edu.cn; zhangwx@sdu.edu.cn; or Xia Zhang, Department of Molecular Biology, Qingdao Vland Biotech Inc., Qingdao, China, E-mail, zhangx@vlandgroup.com.

Running title: Lae1 regulates peptaibols production

Supplemental Data

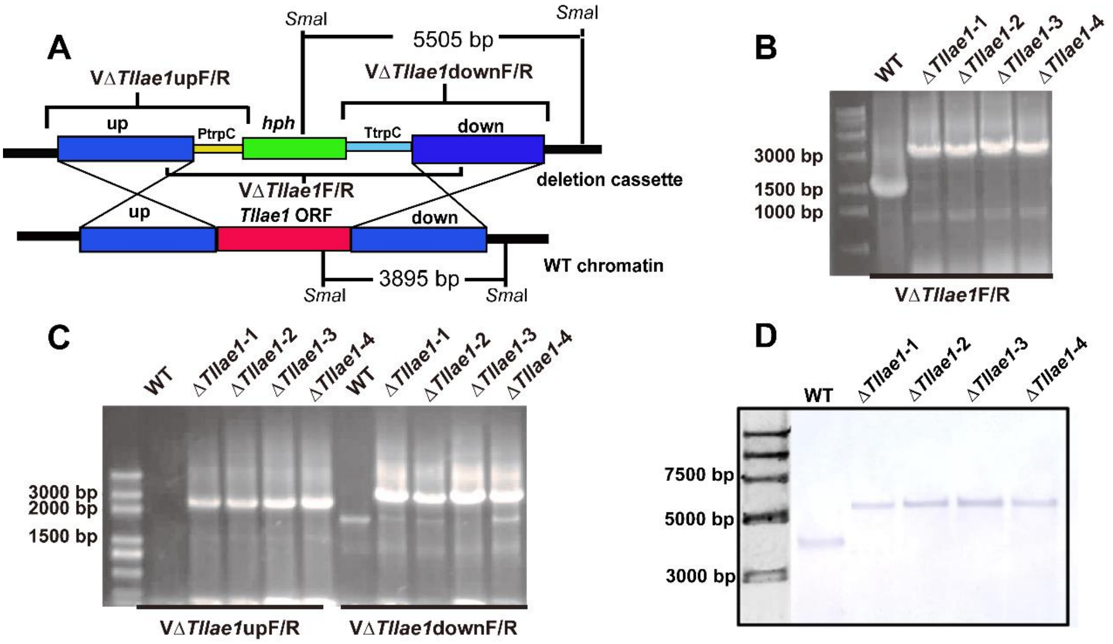

Figure S1. Verification of correct DNA integration in Δ*Tllae1* transformants. (A) Schematic illustration of the *Tllae1* deletion cassette. (B-C) Verification for the correct integration of the *Tllae1* deletion cassette into the genome of *T. longibrachiatum* SMF2 using PCR with the indicated primer pairs. (D) Verification for the correct integration of the *Tllae1* deletion cassette using southern blot hybridization analysis. Genomic DNA of the WT and Δ*Tllae1* strains are digested by *Sma*I and the 676 bp-fragment downstream of the *Tllae1* gene was used as the probe. All the fragments correspond to the expected size as shown in (A).

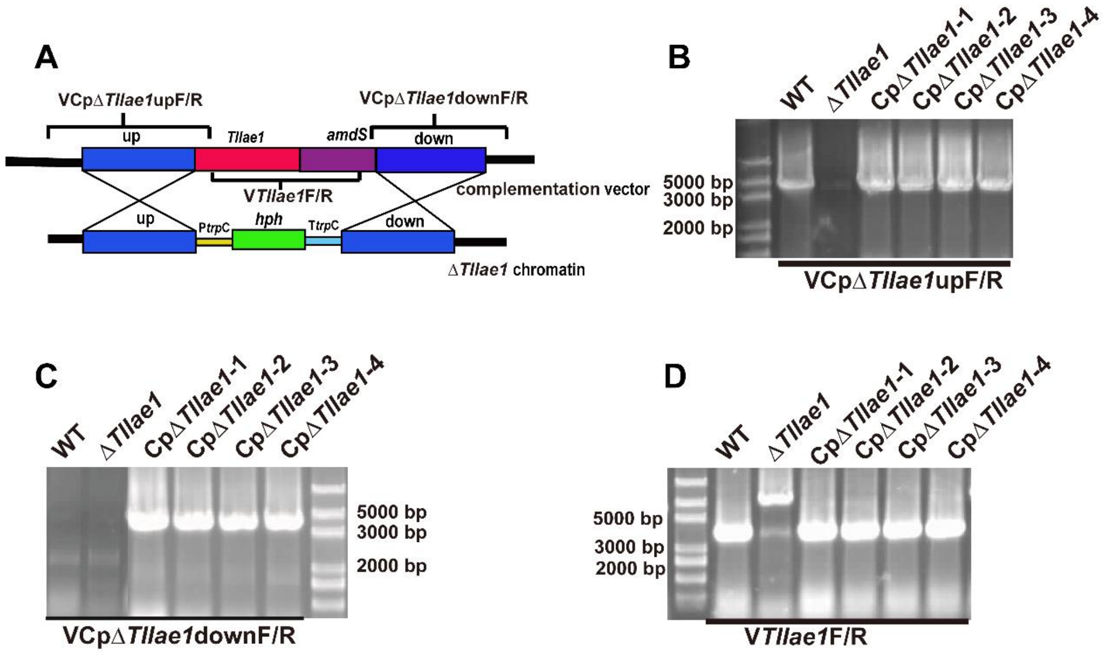

Figure S2. Verification of correct DNA integration in the CpΔ*Tllae1* transformants. (A) Schematic illustration of the *Tllae1* complementation cassette. (B-D) PCR verification for the correct integration of the complementation cassette into the genome of the Δ*Tllae1* strain. All the amplified fragments correspond to the expected size.

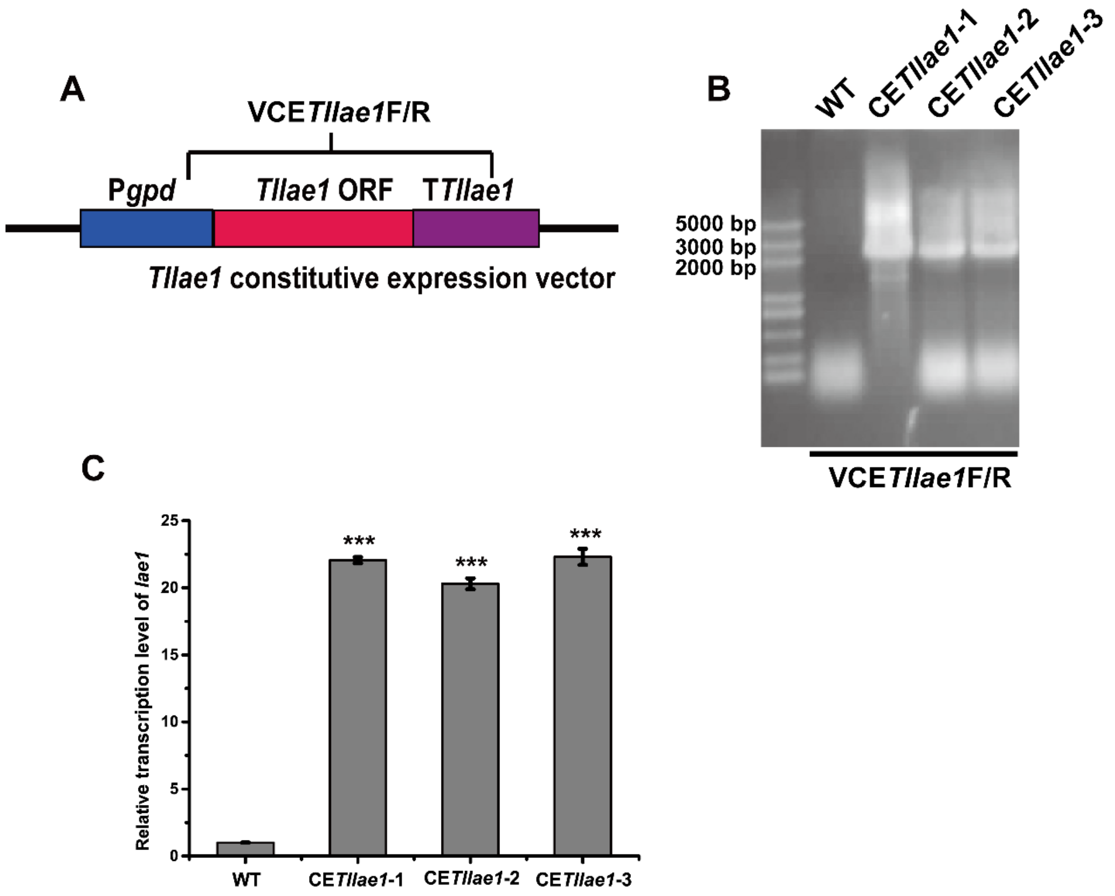

Figure S3. Verification of correct DNA integration and constitutive overexpression of *Tllae1* in the CE*Tllae1* transformants. (A) Schematic illustration of the constitutive expression vector of *Tllae1*. (B) PCR verification for the integration of the constitutive expression vector into the genome of *T. longibrachiatum* SMF2. (C) The relative transcriptional levels of *Tllae1* in WT and CE*Tllae1* strains using quantitative RT-PCR with the primer pair *lae1*-F /*lae1*-R. All the strains were cultured in PDB at 28°C for 48 hours and the mycelia were collected for analyses. The gene *tef1* was used as the control. Values represent the mean of three biological replicates. Error bars are the SD from these replicates. Significant differences (T-test ***P<0.001) were observed in the relative

transcriptional levels of *Tllae1* between WT and CE*Tllae1*.

Table S1. Primers used in this study

Primer	Sequence（5' - 3'）
Δ*Tllae1*upF	TCCTTCGTCTGCTGCTACCGTAC
Δ*Tllae1*upR	CTTCAATATCATCTTCTGTCGACGAACTCTCTCGCCAGATGCAGTG
Δ*Tllae1*downF	CTTGTTTCGGCGTGGGTATGTGACTTGCGTACACAGGCACTTG
Δ*Tllae1*downR	CAGTAGCCTCCAACTGCCGTAC
FhygBF	GTCGACAGAAGATGATATTGAAG
FhygBR	CATACCCACGCCGAAACAAG
CΔ*Tllae1*F	CATTCATACCTTGCCTGACCTTG
CΔ*Tllae1*R	TCCCATCAAGACCAGACTTTGAC
CpΔ*Tllae1*upF	CCCAAGCTTCATTCATACCTTGCCTGACCTTG
CpΔ*Tllae1*upR	GCTCTAGATTTCTATAATGCCTCTAGCCATCATGG
CpΔ*Tllae1*-*amdS*F	GCTCTAGACATCATTGGATAGGCAG
CpΔ*Tllae1*-*amdS*R	GGGGTACCTAGACTGGAAACGCAACCCTG
CpΔ*Tllae1*downF	GGGGTACCGTCATGGTACAGGTCAGACTCCTGCAT
CpΔ*Tllae1*downR	CGGAATTCGCACTGGCAAGCTCATCAGCTTCATCG
*Tllae1*CE-F	CATGCCATGGCCTCTCGAAACGCTCCCAAC
*Tllae1*CE-R	CCCAAGCTTCTAGCTAACCTGTTGCTGTAC
tef1-F	CCACATTGCCTGCAAGTTCGC
tef1-R	GTCGGTGAAAGCCTCAACGCAC
lae1-F	CAAGTCTGCGACTAACAGTTGG
lae1-R	TGAGCTCCTCCTTGTCTATG
